# Breastfeeding mobile application for mothers with gestational diabetes mellitus: designed by mothers and experts

**DOI:** 10.1186/s12889-022-13952-w

**Published:** 2022-08-08

**Authors:** Seungmi Park, Eunju Kwak, Jisan Lee

**Affiliations:** 1 Chungbuk National University, Chungdae-ro 1, Seowon-Gu, Cheongju-si, 28644 Chungcheongbuk-do Korea; 2 Department of Nursing, College of Life and Health Sciences, Hoseo University, 20 Hoseo-ro 79beon-gil, Baebang, Asan, Republic of Korea

**Keywords:** Gestational diabetes mellitus, Breastfeeding, Mobile app, COVID-19, Health promotion, Prevention of complications

## Abstract

**Background:**

Mothers and babies with gestational diabetes have an increased risk of diabetes, obesity, and cardiovascular complications. Breastfeeding is known to help reduce complications in mothers and babies with gestational diabetes. However, the rate of breastfeeding among mothers with gestational diabetes is still low due to various barriers. Therefore, the purpose of this study was to develop a mobile application to improve the breastfeeding barrier of pregnant women with gestational diabetes.

**Methods:**

The Method of App Selection based on Users’ Needs is a method used in designing app structure and user interface by considering user needs. This method was used to develop the Breastfeeding for Gestational Diabetes Mellitus App, reflecting the needs of target users. Four personas were created based on the experiences of four mothers with gestational diabetes mellitus, and these personas’ needs were assessed and prioritized. Two professors and a clinical instructor in women’s health nursing conducted an expert review and revised the contents.

**Results:**

Our “Breastfeeding for Gestational Diabetes Mellitus App” included the following components to promote breastfeeding in mothers with gestational diabetes mellitus: baby growth, breastfeeding records, information about mothers with gestational diabetes mellitus, information about breastfeeding, videos demonstrating breastfeeding methods and breast massage techniques, breastfeeding success stories, a message board, a section for frequently asked questions and answers, and links to breastfeeding education centers.

**Conclusions:**

Use of our App is expected to help prevent complications in mothers with diabetes mellitus and their babies and to promote maternal and child health through improved breastfeeding practices, especially in social distancing situations resulting from COVID-19.

## Background

Gestational diabetes mellitus is diabetes diagnosed during pregnancy. According to the International Diabetes Federation (2019) report, 15.8% of pregnant women worldwide develop diabetes mellitus, and 75–90% develop gestational diabetes mellitus [1]. The number of diabetic patients is expected to continue to increase, and Asia and developing countries are expected to be in the top 10 in 2030 and 2049 [1]. In Korea, the annual prevalence of gestational diabetes mellitus shows a rapidly increasing trend [2]. In the last decade, the prevalence of gestational diabetes mellitus among pregnant women over the age of 35 has increased by an average of 12.7% [3], indicating an increased risk of gestational diabetes mellitus in advanced maternal age.

Gestational diabetes mellitus increases the risks of complications in mothers as well as newborn babies. In mothers, the risks of preterm labor, dystocia of labor, and polyhydramnios are increased [4, 5], and in newborn babies, the fetal death rate and the risks of neurological disorder, heart defect, respiratory distress syndrome, fetal macrosomia, neonatal hypoglycemia, and neonatal jaundice are increased [4, 5]. In addition, the likelihoods of developing type 2 diabetes and childhood obesity are reportedly more than seven times higher in babies of mothers with gestational diabetes mellitus compared to those of healthy mothers [6]. Accordingly, effective prepartum and postpartum management are necessary for maternal and child health.

Breast milk is the most beneficial source of nutrients for newborn babies and infants. A previous study revealed that, compared to formula-fed babies, breastfed babies showed a lower morbidity rate in respiratory disorders, digestive disorders, constipation, eczema, and allergies and were psychologically more stable [7]. The World Health Organization and the United Nations Children’s Emergency Fund recommend exclusive breastfeeding for at least the first 6 months after birth. In Korea, a 2016 survey showed that the rate of exclusive breastfeeding was 95.6% at birth, but it steadily decreased afterward to 18.3% by 6 months. According to a 2018 survey, the rate at 6 months after birth further decreased to 2.3%, showing a sharp decrease [8]. Some reasons for the decreasing rate of breastfeeding in Korea are inability of mothers to initiate breastfeeding at all because of illness or breast pain and due to insufficient milk production [9].

When mothers with gestational diabetes mellitus breastfeed, serotonin production increases, which stimulates beta cells to multiply and reduce oxidative stress in the pancreas [10]. Accordingly, breastfeeding not only helps control blood sugar levels by decreasing insulin resistance in mothers but also lowers, in a long term, the likelihood of the babies to develop childhood obesity, type 2 diabetes mellitus, and cardiovascular disease. Thus, mothers with gestational diabetes mellitus are strongly recommended to breastfeed [11]. However, the rate of breastfeeding is lower in mothers with the condition than in healthy mothers because of physical discomfort due to delayed development of mammary glands and dystocia of labor [12].

Therefore, to prevent complications and promote the health of mothers with gestational diabetes mellitus and their babies, it is necessary to develop programs that encourage effective breastfeeding practices. Currently, the global social distancing policies resulting from the coronavirus disease (COVID-19) pandemic have encouraged non-face-to-face activities. Therefore, mobile app-based breastfeeding intervention programs are expected to be useful for mothers with gestational diabetes mellitus and babies who are vulnerable to infection [13]. A search for breastfeeding-related apps developed so far revealed 251 apps available in the Google Play Store and 91 in the App Store as of May 2022. Most of the available apps were for recording breastfeeding times and breastfeeding instructions for parents. Only 10 apps related to gestational diabetes mellitus were found in Google Play and App Stores, and they were either apps providing dietary information specific to gestational diabetes mellitus or diabetes management apps briefly introducing gestational diabetes mellitus. No app was found for breastfeeding specifically targeting mothers with gestational diabetes mellitus. Consequently, this study aims to develop an easily accessible app-based breastfeeding program to promote maternal and child health through improved breastfeeding health behavior for mothers with gestational diabetes mellitus, which can be applied by those experiencing difficulty in breastfeeding.

## Methods

### Study design

The study was conducted to develop the app structure and user interface (UI) of the Breastfeeding for Gestational Diabetes Mellitus App (BFGDM App). To do so, the Method of App Selection based on User’s Needs (MASUN) was referenced [14]. With MASUN, healthcare professionals who are not app developers can design app structure and UI relatively easily, reflecting user needs. This approach has been used to design apps for women experiencing menstrual cramps [15], for children with obesity and their guardians [16], and for geriatric hospital nurses caring for patients with bed sores [17].

To design the BFGDM App, four mothers with gestational diabetes mellitus who had experience breastfeeding and three researchers in women’s health nursing (a professor of medical informatics and expert in maternity nursing, a professor of maternity nursing, and a clinical lecturer in maternity nursing) participated in the study. In total, the study was conducted with seven participants between January 20 and April 30, 2022.

#### Step 1: App list based on users’ needs

The study received ethical approval from the Chungbuk National University Committee of CBNU-202202-HR-0023. After seeking consent from the participants, the study began. Discussion of the list of app components was conducted by a researcher with experience studying app development using a user-based method (MASUN). To reflect the needs of actual users, discussion also included breastfeeding mothers with gestational diabetes who had previous successful breastfeeding experiences and the researchers as an expert group. The mothers who had participated in the researchers' previous study [18] expressed their intention to participate in the future study, and a focus group interview was conducted with participants who were successful in breastfeeding for 6 months. The interview generated a list of elements to include when designing a breastfeeding app for pregnant women with gestational diabetes, including information and methods necessary for breastfeeding success and methods to solve difficulties during breastfeeding. Based on the derived opinions, the contents were selected together with researchers who specialized in maternal nursing, and App User and App User’s problems were selected.

#### Step 2: Brainstorming and mind mapping

Concept mapping was used to systematically and flexibly organize complex problems [19]. Group concept mapping is a collaborative process that is strict, scientifically reliable, and an important practical tool. It has been widely used in health management projects [19–21]. Of various available concept mapping methods, group brainstorming and mind mapping were used in this study. Brainstorming is a process that helps generate ideas and gain an in-depth understanding of a topic, and mind mapping is a tool to show a core idea and new ideas derived from the core idea [22].

#### Step 3: Development of persona and scenario

Conventionally, a systematic approach without a model tailored to a particular user group has been employed in designing smartphone apps. However, using profiles and personas has become a more valuable approach [23]. Personas help technology developers gain an in-depth understanding of diverse target users, and they are an important feature in user-centered design [24]. They provide a guideline to technology developers in creating a software interface for users with specific health needs [23]. In this study, four personas and scenarios were created, and the app functions needed by each persona were organized into a list.

#### Step 4: Needs list and priority consideration

Based on the personas and scenarios developed in Step 3, user needs were organized into categories. The categories served as the criteria for the menu and functions to be included in designing the app structure and UI.

#### Step 5: App structure and UI design

The designs for the app structure and UI of the BFGDM App were drafted by reflecting the user needs derived in Step 4. Pen and paper were used in drafting the designs so that they would be simple and easy for the researchers.

Four mothers with gestational diabetes mellitus who had experience breastfeeding and two professors and one clinical instructor in women’s health nursing reviewed and revised the draft designs (a total of seven participants).

#### Step 6: Final setting up of “Mental Healing App” app structure and UI

The app structure and UI of the BFGDM App were derived from the researchers’ expert review and revision. A mobile UI designer finalized the designs.

## Results

### *Step 1*: *App list based on users’ needs*

The group of gestational diabetic mothers and researchers selected supporters for mothers with gestational diabetes based on the opinions shared through two interviews and discussions and attempted to propose mobile breastfeeding care for them.

### Step 2: Brainstorming and mind mapping

**Fig. 1:**
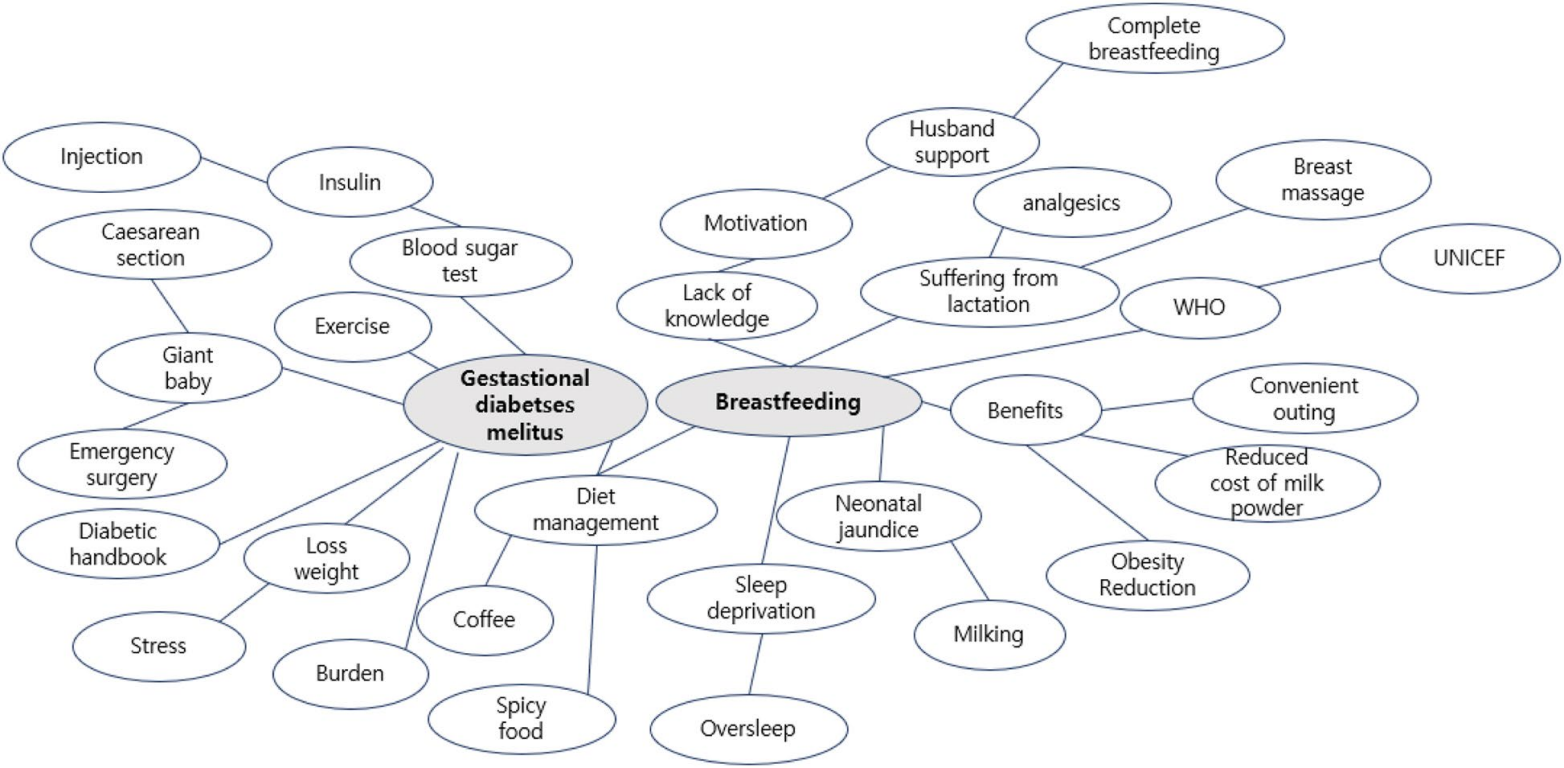
Mind map on gestational diabetes mellitus and breastfeeding

In this stage, seven researchers organized related problems and target ideas selected through group brainstorming and mind mapping conducted twice by a group of four women with gestational diabetes and three researchers (Fig. 1).

### Step 3: Development of persona and scenario

#### Persona A – Mother with gestational diabetes mellitus

She lives with her husband in Cheonan, South Chungcheong Province. Her parents and parents-in-law live nearby. Currently, she is taking a leave of absence from a job due to pregnancy. This is her first pregnancy, and she is diagnosed with gestational diabetes mellitus. Housewife. Name: Jin-Ah Kim (age 38, female).

### Subpersona A– Support provider

He lives in Cheonan, Sough Chungcheong Province, and works as a public official. He helps the pregnant wife and assists in child caring. The husband of Jin-Ah Kim. Name: Hyeong-Gyu, Park (age 40, male).

#### Persona B – Mother with gestational diabetes mellitus

She lives with her husband and their first child in Suwon, Gyeonggi Province. Diagnosed with gestational diabetes mellitus during the second pregnancy. Housewife. Name: Na-Hyeon Kim (age 33, female).

### Subpersona B– Support provider

She lives in Suwon, Gyeonggi Province. She and Na-Hyeon were clients at the same postnatal care center during Na-Hyeon’ first childbirth. She breastfed her baby successfully. Housewife. Name: Mi-Ran Choi (age 35, female).

### Step 4: Needs list and priority consideration

1) Needs derived for Persona A were as follows.

- Baby’s height and weight, normal height and weight, growth chart
- Breastfeeding date and time, the side of the breast used, daily frequency of breastfeeding, and breastfeeding statistics
- Features of breastmilk of mothers with gestational diabetes mellitus, advantages of breastfeeding for mothers with the condition, and information regarding breastfeeding education centers
- Techniques of breastfeeding and breast massage
- Community to share breastfeeding success stories

2) Needs derived for Persona B were as follows.

- Information regarding postpartum management in mothers with gestational diabetes mellitus
- Comparison of the baby’s height and weight with normal height and weight
- Information about the baby’s growth month by month against normal growth
- Brief records of the side of the breast used and the beginning and end of a breastfeeding session
- Information search regarding symptoms of breast problems and management
- Recommendations of breastfeeding and epilogues on breastfeeding posted on a message board
- Articles containing information regarding gestational diabetes mellitus and breastfeeding and advantages of breastfeeding for mothers with gestational diabetes mellitus
- Frequency of breastfeeding by date; statistics; and graphs showing daily, weekly, and monthly average durations of breastfeeding sessions

### Step 5: App structure and UI design

Researchers developed needs lists and determined priorities for the four personas and summarized the structure and components of the BFGDM-App in a table [Table 1]. The BFGDM-App included baby development, recording, information, statistics and graphs, community, and professional institution. Baby development covered the baby’s name, height, weight, growth and development, and developmental information by monthly age. Recording covered the date, number of breastfeeding sessions, breastfeeding time, location of lactating breast, and number of breast massages. Information included characteristics of breast milk in mothers with gestational diabetes, benefits of breastfeeding in mothers with gestational diabetes, breastfeeding success stories, breastfeeding method (video), and breast trouble management. Statistics and graphs included baby's statistics, breastfeeding statistics, and statistics on the number of breast massages performed. Community included frequently asked questions and answers and message boards, and Professional Institutions included information from breastfeeding specialist organizations.

**Table 1:** List of priority-based user needs for BFGDM-Apps

Baby Development	Recording	Information	Statistics and graphs	Community	Professional Institutions
Name	Date	Characteristics of breast milk in gestational diabetic mothers	Baby’s statistics (height, weight)	Frequently as ask questions and answers	Information from breastfeeding specialist organizations (URL)
Height	Number of breastfeeding	Benefits of breastfeeding in gestational diabetic mothers	Breastfeeding statistics (Daily, monthly)	Board	
Weight	Breastfeeding time: Stop-watch	Breastfeeding success stories	Statistics of the number of breast- massage performed		
Growth and Development	Location of lactating breast	Breastfeeding method (video)			
Developmental information by monthly age	Number of breast massages	Breast trouble management			

### Step 6: Final setting up of “Mental Healing App” app structure and UI

**Fig. 2:**
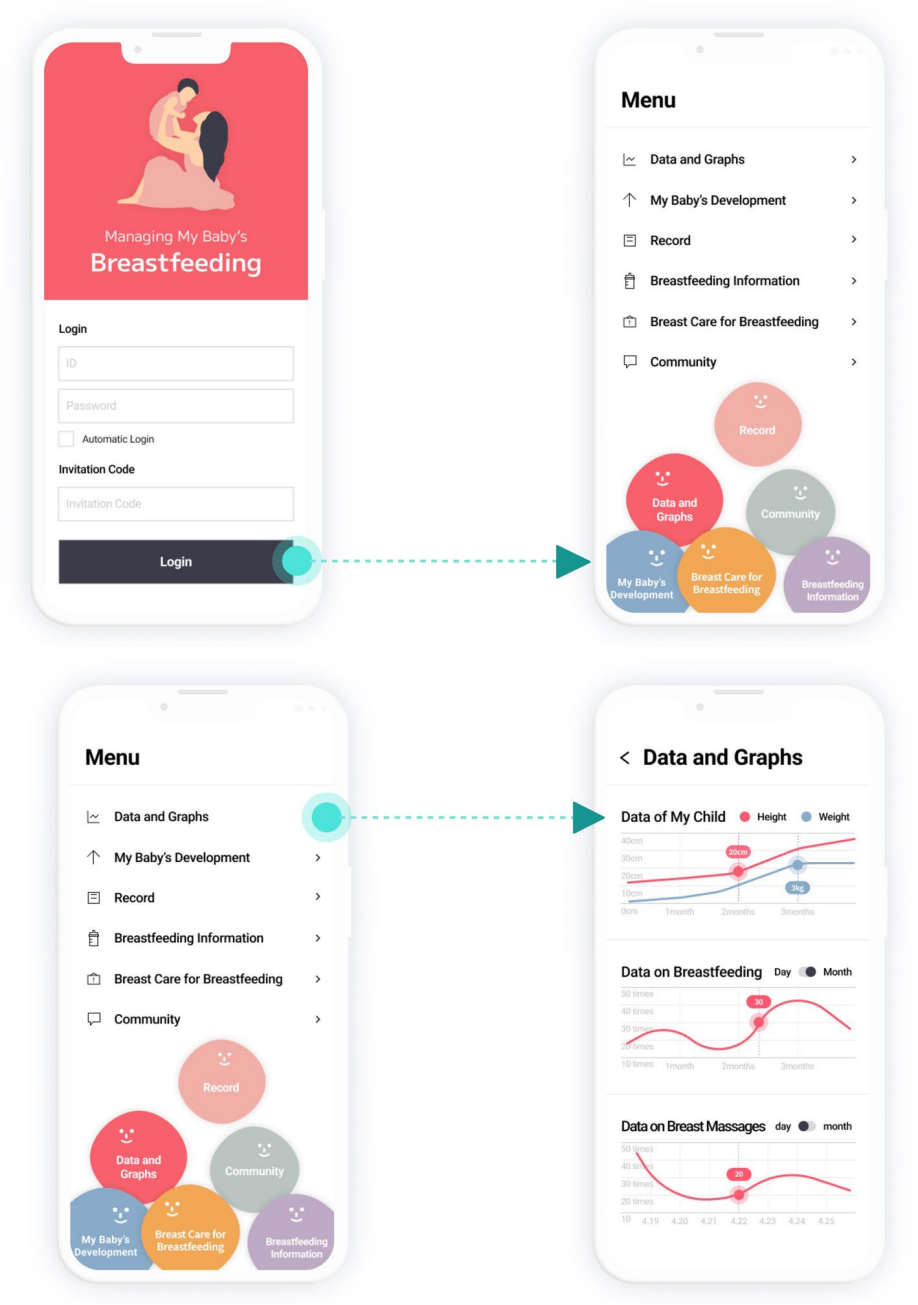
BFGDM-App UI and user flow- menu, data and graphs

**Fig. 3:**
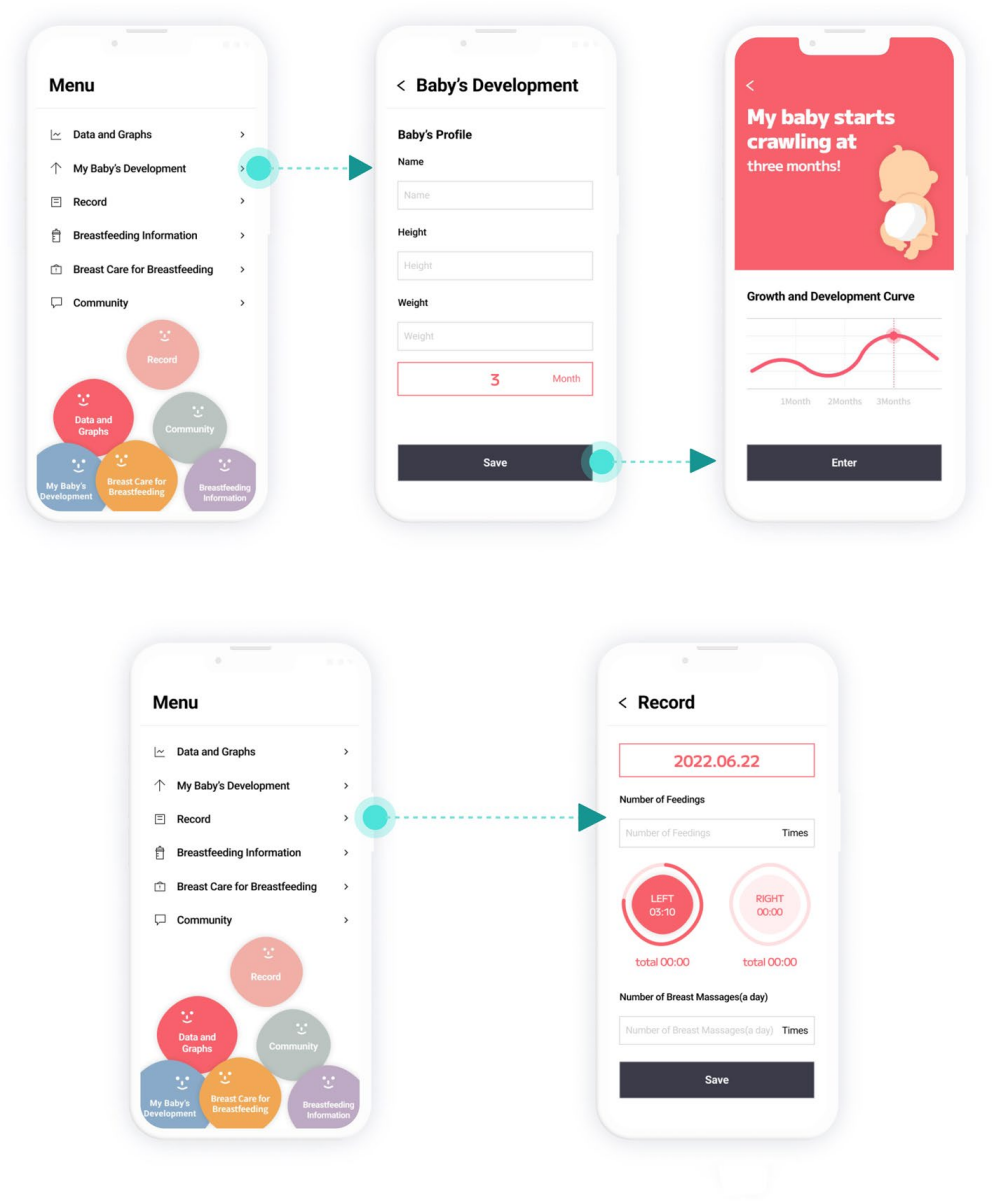
BFGDM-App UI and user flow- baby’s development, record

**Fig. 4:**
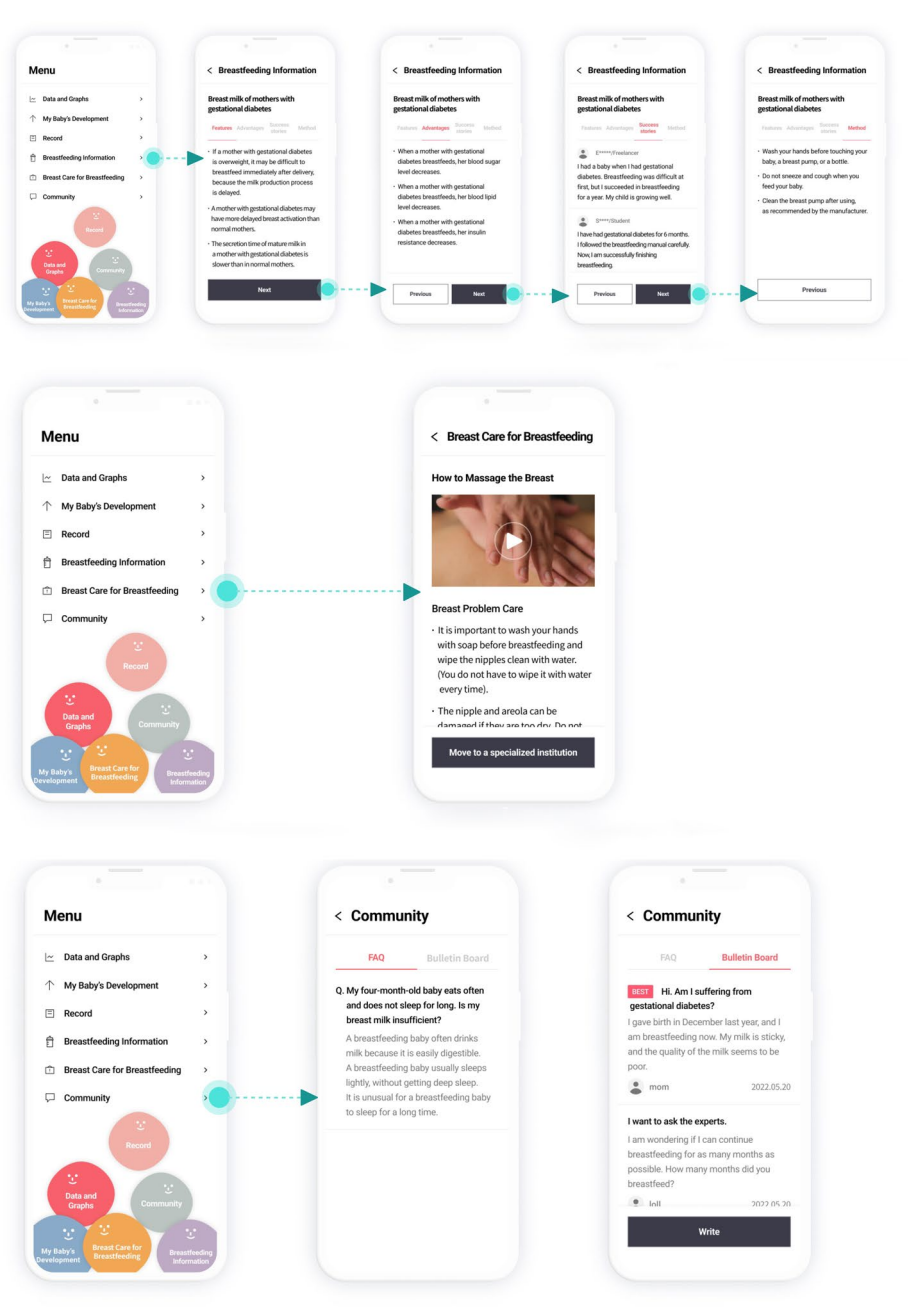
BFGDM-App UI and user flow- breastfeeding information and care, community

After the UI design was drafted based on the summary table, the researchers conducted an expert review. Based on the expert review and revisions, the draft design was revised, and the app structure and UI of the BFGDM App were derived (Figs. 2, 3 and 4). Thereafter, a total of four rounds of discussion were held with the mobile UI designer to finalize the design of the UI. Specific UI design features were as follows. The first screen consisted of login and invitation code areas, and the menu screen was designed such that a click on a submenu would open the corresponding menu. The screen for baby growth consisted of areas in which the user could record the baby’s height and weight. A click on age by month showed a normal growth chart to check the baby’s current growth status against the norms. The data screen was designed to show the daily frequency of breastfeeding recorded via a stopwatch and a link to breastfeeding statistics so that the user could check the results. The screen for breastfeeding information was designed to show written text regarding the features of breast milk for mothers with gestational diabetes mellitus, advantages of breastfeeding for mothers with the condition, breastfeeding success stories, and videos showing breastfeeding methods and breast massage techniques to provide a guide for breast management during breastfeeding. Last, the community screen was designed so that the user could search for answers to fundamental questions regarding breastfeeding and communicate with one another through a message board.

## Discussion

Mothers with gestational diabetes mellitus and their babies have increased risks for diabetes, obesity, and cardiovascular complications [4, 5], and breastfeeding is reported to help lower these risks [10, 11]. However, due to various barriers, the rate of breastfeeding is low among mothers with gestational diabetes mellitus [12]. This study is significant because, in the current situation in which people adhere to social distancing due to COVID-19, a mobile application design was developed to help mothers with diabetes mellitus who are vulnerable to infection and have difficulty breastfeeding. As of May 2022, apps for breastfeeding and management of gestational diabetes mellitus were developed separately, and, thus, mothers with gestational diabetes mellitus must use at least two apps to obtain help with breastfeeding. The development of the BFGDM App allows mothers with the condition to use a single app, which increases accessibility and convenience.

To promote breastfeeding in mothers with diabetes mellitus, the application design was developed by referring to MASUN [15]. In addition, to improve the app’s usability, the app structure and UI were implemented based on the experiences and needs of four mothers with gestational diabetes mellitus and also on user needs derived by three researchers in women’s health nursing (two professors and a clinical instructor), who were not app developers, using the method specified in MASUN [25]. The methodology of involving end users of the to-be-developed app in the process of implementing the UI and creating a draft design before finalizing it has also been utilized in previous studies [17, 25].

The method of this study can be used by researchers who plan to create a mobile app UI for target users with a small number of study participants over a relatively short time [17]. The BFGDM App was developed to promote breastfeeding in mothers with gestational diabetes mellitus with focus on addressing their questions and difficulties and stimulating community activity to increase the motivation for breastfeeding by providing overall information regarding breastfeeding, how to breastfeed, a breastfeeding journal, breast management techniques, and breastfeeding questions and answers.

Despite delayed development of mammary glands and physical discomfort during labor [12], mothers with diabetes mellitus who breastfed stated that, with short-term suffering, they were able to reduce the feeling of guilt by providing their babies with the best nutrition. They enumerated specific knowledge and emotional support as success factors for breastfeeding [26]. This is consistent with a finding derived from the user needs survey conducted in this study, i.e., informational needs. To provide breastfeeding-specific information, the BFGDM App was designed to show the features of breastmilk for mothers with diabetes mellitus, advantages of breastfeeding to mothers with the condition, successful breastfeeding stories, breastfeeding methods, breast massage techniques, and breast problem management in the breastfeeding information screen. It is believed that this detailed information will help app users acquire the knowledge they need when breastfeeding their baby. In particular, videos demonstrating breastfeeding and breast management methods were included in the app to help users learn the techniques easily. Additionally, the app was designed to make it easy for users to acquire specialized knowledge and learn how to manage breast problems immediately by providing contact information of breast management education centers.

The data screen consisted of recordings about breastfeeding sessions and baby growth. The design components were created by combining the findings derived from the user needs survey and the functions of existing parenting apps (Babytime: over 1 million downloads, a star rating of 4.9; Parenting Diary: over 500,000 downloads, a star rating of 4.8). The components are expected to be helpful to app users in monitoring the status of breastfeeding and the baby’s growth as well as self-management. The advantages of mobile apps include accessibility and self-monitoring capacity. Hence, it is believed that the BFGDM App will self-motivate users to continue breastfeeding. The recording of breastfeeding sessions via a stopwatch was developed with a focus on user convenience, and the function requires only a simple operation. The benefits of using this function are that users can check the previous breastfeeding time and the side used to track the interval between breastfeeding sessions and alternate the breast.

It has been reported that the breastfeeding practice of mothers with diabetes mellitus is influenced by their breastfeeding experience and self-efficacy [9]. The BFGDM App was designed to help increase breastfeeding self-efficacy in mothers with gestational diabetes mellitus by providing an app that uses a message board to share breastfeeding experiences on the community screen and successful breastfeeding stories on the breastfeeding information screen as well as to inspire users to believe that they can also breastfeed successfully. Therefore, it is expected that, particularly in the COVID-19 situation, in which non-face-to-face interaction is encouraged, the use of the BFGDM App will provide emotional support, increased self-efficacy, self-management practices, and experiences of other mothers with the same condition to promote breastfeeding practice among mothers with diabetes mellitus.

The BFGDM App is structured for users to easily obtain information about breastfeeding through text, graphs, and videos; provides features that facilitate sharing of breastfeeding experiences; and provides links to breast management education centers. This app will be an important tool in promoting breastfeeding. It can be used to improve the knowledge, experience, and self-efficacy in breastfeeding of mothers with gestational diabetes mellitus and minimize the impact of the disease on breastfeeding practice among them [9]. In the future, a follow-up study should be conducted to examine the effects of BFGDM App by assessing breastfeeding knowledge, intention, and success in mothers with gestational diabetes mellitus who use the app.

## Conclusions

Use of the breastfeeding app developed through this study is expected to help breastfeeding mothers with gestational diabetes in the context of social distancing due to COVID-19. In addition, the app, which can be used for breastfeeding by mothers with gestational diabetes, is designed with input from pregnant women with gestational diabetes who previously succeeded in breastfeeding, professors majoring in medical information and maternal nursing, maternal nursing professors, and researchers designing apps for health management. In particular, the five steps used in the app design in this study reflect the needs of study participants and can be utilized by pregnant women with gestational diabetes who are not app developers. Finally, the structure and UI of the app visualized as a user flow can be used as a basis for the future development of the Breastfeeding for Gestational Diabetic Mothers app. Research is needed to confirm the app’s effectiveness.

## Data Availability

The datasets used and/or analyzed during the current study are available from the corresponding author on reasonable request.

## Abbreviations

MASUN: The Method of App Selection based on Users' Needs
UI: User interface
BFGDM: Breastfeeding for Gestational Diabetes Mellitus App
COVID-19: Coronavirus disease
